# Serological immune response against ADAM10 pro-domain is associated with favourable prognosis in stage III colorectal cancer patients

**DOI:** 10.18632/oncotarget.11181

**Published:** 2016-08-10

**Authors:** Sheila María Álvarez-Fernández, Marco Barbariga, Luca Cannizzaro, Carlo Vittorio Cannistraci, Laura Hurley, Alan Zanardi, Antonio Conti, Francesca Sanvito, Anna Innocenzi, Nicolò Pecorelli, Marco Braga, Massimo Alessio

**Affiliations:** ^1^ Proteome Biochemistry, IRCCS-San Raffaele Scientific Institute, Milan, Italy; ^2^ Pathology, IRCCS-San Raffaele Scientific Institute, Milan, Italy; ^3^ Department of Surgery, Vita-Salute San Raffaele University, Milan, Italy; ^4^ Biomedical Cybernetics Group, Biotechnology Center (BIOTEC), Center for Molecular and Cellular Bioengineering (CMCB), Technische Universität Dresden, Dresden, Germany; ^5^ Translational Neurology group, Wallenberg Neuroscience Center, BMC, Lund, Sweden; ^6^ Systems Biology Center, Faculty of Medicine, Chulalongkorn University, Bangkok, Thailand; ^7^ Wayne State University, School of Medicine, Cancer Biology PhD Program, Detroit, Michigan, USA

**Keywords:** ADAM10 metalloprotease, colorectal carcinoma, immuno-proteomics, autoantibodies, prognosis

## Abstract

A humoral immune response against aberrant tumor proteins can be elicited in cancer patients, resulting in the production of auto-antibodies (Abs). By serological proteome analysis we identified the surface membrane protein ADAM10, a metalloproteinase that has a role in epithelial-tumor progression and invasion, as a target of the immune response in colorectal cancer (Crc). A screening carried out on the purified protein using testing cohorts of sera (Crc patients *n* = 57; control subjects *n* = 39) and validation cohorts of sera (Crc patients *n* = 49; control subjects *n* = 52) indicated that anti-ADAM10 auto-Abs were significantly induced in a large group (74%) of colon cancer patients, in particular in patients at stage II and III of the disease. Interestingly, in Crc patients classified as stage III disease, the presence of anti-ADAM10 auto-Abs in the sera was associated with a favourable follow-up with a significant shifting of the recurrence-free survival median time from 23 to 55 months. Even though the ADAM10 protein was expressed in Crc regardless the presence of auto-Abs, the immature/non-functional isoform of ADAM10 was highly expressed in the tumor of anti-ADAM10-positive patients and was the isoform targeted by the auto-Abs. In conclusion, the presence of anti-ADAM10 auto-Abs seems to reflect the increased tumor expression of the immunogenic immature-ADAM10 in a group of Crc patients, and is associated with a favourable prognosis in patients at stage III of the disease.

## INTRODUCTION

In cancer, the host's immune system acts to eliminate cells expressing qualitatively and/or quantitatively aberrant proteins that can be recognised by both T and B cells. Auto-Abs fostered by anti-tumour immunity can be exploited for the identification of tumoral antigens, and the presence of auto-Abs can be used as a biomarker [1–5]. Serological proteome analysis (SERPA) exploits the reactivity of sera from cancer patients to screen the tumor proteome resolved by two-dimensional electrophoresis (2DE) and has been developed as an approach for the discovery of markers in malignancies [1, 2, 5]. Some of the antigenic proteins identified by immunoproteomics, including our previous works, have provided biomarker candidates that may be of clinical use concerning diagnosis and prognosis, and may represent new candidate antigens for cancer immunotherapy as a specific cellular immune-response can be induced toward these antigens [6–14]. A benefit of the SERPA approach is the ability to highlight the reactivity against specific protein isoforms and post-translational modifications that can be aberrantly expressed by tumour cells [10, 15]. This approach has been successfully applied to several different types of cancer (reviewed in [2, 5]) including colorectal cancer (Crc) [15–18]. Nevertheless, Crc remains poorly characterized for its antigen content. In previous studies, we applied the SERPA approach to characterize the humoral immune response in Crc patients identifying immunogenic proteins specifically expressed or over-expressed in tumor cells [15, 18]; however no surface membrane proteins were found to be immunogenic. We hypothesized that immunoreactivity against membrane proteins failed to be detected due to their poor presence in the total cell lysates that were used for the proteome screening. In order to identify auto-Abs directed against putative membrane antigens, in this study we applied a membrane enrichment procedure coupled with SERPA. This approach allowed us to identify ADAM10 (A Disintegrin And Metalloproteinase 10) as a membrane protein able to elicit a specific humoral response in Crc patients. ADAM10 enzymatic functions have been reported to play a role in epithelial tumor progression and invasion [19], therefore, we further investigated the immunological reactivity of Crc patients toward this molecule performing a serological screening against the purified protein. We found that in Crc patients at stage III of the disease the presence of anti-ADAM10 auto-Abs is associated with a favourable prognosis, and that the anti-ADAM10 serological reactivity reflects the increased expression of the immature non-functional ADAM10 isoform in the tumor cells of the patients. The increase of the ADAM10-immature isoform expression is likely to be immunogenic and to contribute to the reduction of net ADAM10 activity, which is a beneficial condition that may be instrumental in the limitation of cancer progression.

## RESULTS

### Membrane protein ADAM10 is a target of auto-Abs in colorectal cancer

**Figure 1:**
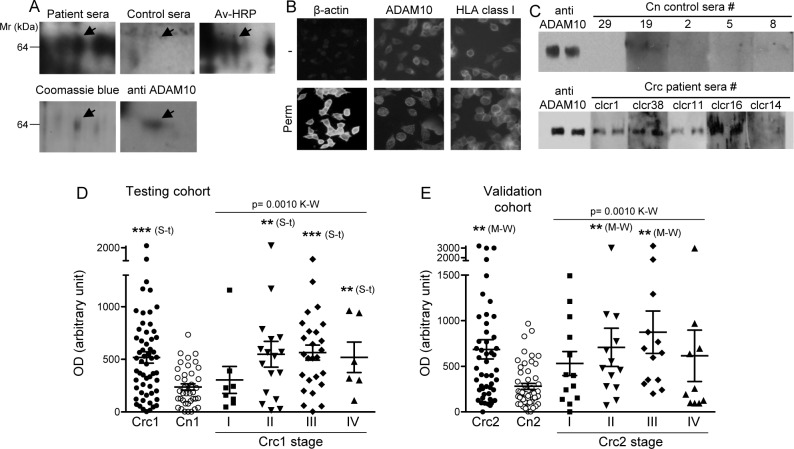
ADAM10 surface protein induces serological immune-response in Crc patients **A.** Serological reactivity of Crc patient pool of sera and control pool of sera against the biotinylated protein spot (Av-HRP) that, from the preparative 2DE gel (Coomassie blue), yielded the MS identification of ADAM10; the identity was confirmed by reactivity of an anti-ADAM10 Ab with the same spot. **B.** Surface expression of ADAM10 in the LS180 Crc cell line. Anti-ADAM10 immunofluorescence reactivity is present on both permeabilized and non-permeabilized cells. Anti-HLA-class I and anti- ß-actin reactivities were used as controls for surface and intracellular expressed proteins, respectively. **C.** Reactivity of Crc patients and control subjects (Cn) sera against purified ADAM10; anti-ADAM10 Ab reactivity was used for signal normalization. **D.**-**E.** Quantitative analysis of serological reactivity reported as normalized OD (mean +/− SEM of 3 experiments in duplicate). **D.** Testing cohorts Crc1, *n* = 57; Cn1, *n* = 39; Crc1-stage I *n* = 8, stage II *n* = 17, stage III *n* = 26, stage IV *n* = 6. **E.** Validation cohorts Crc2, *n* = 49; Cn2, *n* = 52; Crc2-stage I *n* = 13, stage II *n* = 13, stage III *n* = 13; stage IV *n* = 10. Statistical analysis was performed by either student-t (S-t) test or Mann-Whitney (M-W) test and non parametric analysis of variance by Kruskal-Wallis (K-W). (*** = *p* < 0.0001; ** = *p* < 0.01).

An enrichment in the biotinylated surface proteins was found in the fraction of protein extracted from LS180 cells bound to the avidin-affinity-column as inferred by hybridization with HRP-streptavidin on 2DE-resolved protein (Supplemental Materials SM-Figure 1A), and as confirmed by western blot (WB) reactivity for membrane proteins (SM-Figure 1B). In order to determine whether sera from Crc patients identified in the previous study as immunoreactive for intracellular antigens [15] might also contain auto-Abs specific for membrane antigens, we used as a discovery tool a pool of six of these sera to test the reactivity on the 2DE-membrane tumor proteome. As a control a pool of healthy subject sera was used. Several spots were reactive and the corresponding proteins have been identified by mass spectrometry (MS) analysis (not shown); among these we focused our attention on ADAM10, a surface metalloproteinase. The protein spot that yielded the MS-identification (entry name: ADA_10HUMAN; accession-# UniProtKB: O14672; sequence coverage, 16%; fragmented peptides, 10; Mascot score, 206), was recognized by the pool of Crc patients sera but not by the pool of control subjects sera, and it was reactive with avidin-HRP proving its surface expression (Figure 1A). ADAM10 identification was confirmed by the WB reactivity of an anti-ADAM10 Ab with the spot corresponding to the preparative gel spot which yielded ADAM10 identification by MS (Figure 1A). The LS180 cell surface expression of ADAM10 was validated by immunofluorescence analysis that showed the anti-ADAM10 reactivity both in non-permeabilized and permeabilzed cells (Figure 1B).

### Sera from Crc patients contain anti-ADAM10 auto-Abs

A serological screening on recombinant ADAM10 resolved by SDS-PAGE was performed using the sera collected in our previous study as testing cohorts (patients Crc1, *n* = 57; controls Cn1, *n* = 39) [15] (Table 1). The analysis showed that the immunoreactivity against ADAM10 was higher in patient sera compared to control sera (Crc1 *vs*. Cn1, *p* = 0.0001) (Table 2; Figure 1C, 1D) (optical density for each serum is reported in Supplemental Material SM-Table 1 and 3). In particular, the higher immunoreactivity was found associated to Crc patients at stage II and III of the disease (*p* = 0.0013 and *p* < 0.0001, respectively by student-t test) when different stages were considered independently (Table 2 and Figure 1D). Distinctions were also found by nonparametric analysis of variance (*p* = 0.0010, Kruskal-Wallis test), with post analysis test significant for Crc1 patients at stage III (*p* < 0.01) compared to controls.

**Table 1:** Summary of the demographic and clinical features of the analyzed populations

Testing cohorts													
		Crc1		Stage I		Stage II		Stage III		Stage IV		Cn1	
Characteristics		(*n* = 57)		(*n* = 8)		(*n* = 17)		(*n* = 26)		(*n* = 6)		(*n* = 39)	
		*n*	%	*n*	%	*n*	%	*n*	%	*n*	%	*n*	%
**Sex**													
	male	32	*56*	6	*75*	8	*47*	15	*58*	3	*50*	15	*38*
	female	25	*44*	2	*25*	9	*53*	11	*42*	3	*50*	24	*62*
**Age (years)**													
	mean	67	*-*	65	*-*	68	*-*	67	*-*	65	*-*	64	*-*
	range	44-90	*-*	53-83	*-*	51-84	*-*	44-90	*-*	57-74	*-*	27-91	*-*
**Tumor stage**													
	I	8	*14*	8	*100*								
	II	17	*30*			17	*100*						
	III	26	*46*					26	*100*				
	IV	6	*10*							6	*100*		
**Tumor grading**													
	n.r.	7	*12*	1	*13*	-	*-*	3	*11*	3	*50*		
	1	3	*5*	3	*37*	-	*-*	-	*-*	-	*-*		
	2	39	*69*	4	*50*	14	*82*	21	*81*	-	*-*		
	3	8	*14*	-	*-*	3	*18*	2	*8*	3	*50*		
**Tumor primary site**													
	colon	55	*96*	7	*88*	17	*100*	25	*96*	6	*100*		
	rectum	2	*4*	1	*12*	-	*-*	1	*4*	-	*-*		
**Surgery**													
	yes	57	*100*										
	no	-	*-*										
**Therapy**													
**neoadjuvant (CT/RT)**	yes	50	*88*	1	*12*	17	*100*	26	*100*	6	*100*		
	no	7	*12*	7	*88*	-	*-*	-	*-*	-	*-*		
**adjuvant (CT)**	yes	57	*100*										
	no	-	*-*										

Validation cohorts													
		Crc2		Stage I		Stage II		Stage III		Stage IV		Cn2	
Characteristics		(*n* = 49)		(*n* = 13)		(*n* = 13)		(*n* = 13)		(*n* = 10)		(*n* = 52)	
		*n*	%	*n*	%	*n*	%	*n*	%	*n*	%	*n*	%
**Sex**													
	male	26	*53*	7	*54*	6	*46*	6	*46*	7	*70*	22	*42*
	female	23	*47*	6	*46*	7	*54*	7	*54*	3	*30*	30	*58*
**Age (years)**													
	mean	68	*-*	68	*-*	74	*-*	66	*-*	62	*-*	64	*-*
	range	37-96	*-*	41-85	*-*	52-96	*-*	47-80	*-*	37-75	*-*	32-92	*-*
**Tumor stage**													
	I	13	*27*	13	*100*								
	II	13	*27*			13	*100*						
	III	13	*27*					13	*100*				
	IV	10	*19*							10	*100*		
**Tumor grading**													
	n.r.	1	*2*	-	*-*	-	*-*	-	*-*	1	*10*		
	1	5	*10*	4	*31*	-	*-*	1	*8*	-	*-*		
	2	32	*65*	7	*54*	10	*77*	7	*54*	8	*80*		
	3	11	*23*	2	*15*	3	*23*	5	*38*	1	*10*		
**Tumor primary site**													
	colon	38	*78*	9	*69*	13	*100*	8	*62*	8	*80*		
	rectum	11	*22*	4	*31*	-	*-*	5	*38*	2	*20*		
**Surgery**													
	yes	49	*100*										
	no	-	*-*										
**Therapy**													
**neoadjuvant (CT/RT)**	yes	40	*82*	4	*31*	13	*100*	13	*100*	10	*100*		
	no	9	*18*	9	*69*	-	*-*	-	*-*	-	*-*		
**adjuvant (CT)**	yes	49	*100*										
	no	-	*-*										

Crc= colorectal cancer; Cn= control subjects; n.r.= not reported; CT= chemotherapy; RT= radiotherapy

**Table 2:** Anti-ADAM 10 serological reactivity

		normalized OD	95% CI	vs Cn
Testing cohorts	Crc1 (*n* = 57)	518.2 se 54.1	409.8-626.6	*p* = 0.0001 (S-t)
	Stg I (*n* = 8)	304.0 se 127.8	1.8-606.2	ns (M-W)
	Stg II (*n* = 17)	548.2 se 122.3	288.8-807.5	*p* = 0.0013 (S-t)
	Stg III (*n* = 26)	564.3 se 71.2	417.7-710.9	*p* < 0.0001 (S-t)
	Stg IV (*n* = 6)	519.3 se 145.1	146.5-892.2	*p* = 0.0040 (S-t)
	Cn1 (*n* = 39)	237.5 se 29.4	177.9-297.1	
Validation cohorts	Crc2 (*n* = 49)	686.1 se 105	474.9-897.2	*p* = 0.0003 (M-W)
	Stg I (*n* = 13)	513.3 se 131.5	244.8-817.9	ns (M-W)
	Stg II (*n* = 13)	707.7 se 209.9	250.3-1165	*p* = 0.0057 (M-W)
	Stg III (*n* = 13)	873.7 se 232.6	367-1380	*p* = 0.0002 (M-W)
	Stg IV (*n* = 10)	615.3 se 280.9	20.2-1251	ns (M-W)
	Cn2 (*n* = 52)	279.9 se 32.1	215.5-344.4	

Crc= colorectal cancer; Cn= control subjects; Stg I-II-III-IV= stages of colon-carcinoma patients; se= standard error; OD= optical density; S-t= student-t test; M-W= Mann-Whitney test; 95% CI= 95% confidence interval.

The difference in immunoreactvity was confirmed using the validation cohorts of sera collected from Crc patients (Crc2, *n* = 49) and healthy subjects (Cn2, *n* = 52) (*p* = 0.0003) (Table 2 and Figure 1E) (optical density for each serum is reported in SM-Table 2 and 4). Also in these cohorts, patients at stage II and III of the disease showed greater immunoreactivity against ADAM10 than controls (*p* = 0.0057 and *p* = 0.0002, respectively by Mann-Whitney; and *p* = 0.0014 by Kruskal-Wallis test, with post test analysis *p* < 0.01 for stage III patients) (Table 2 and Figure 1E). These data suggested that the immunoreactivity against ADAM10 might be a feature able to distinguish a large group of Crc patients, in particular those at stage II and even more significantly those at stage III of the disease.

**Figure 2:**
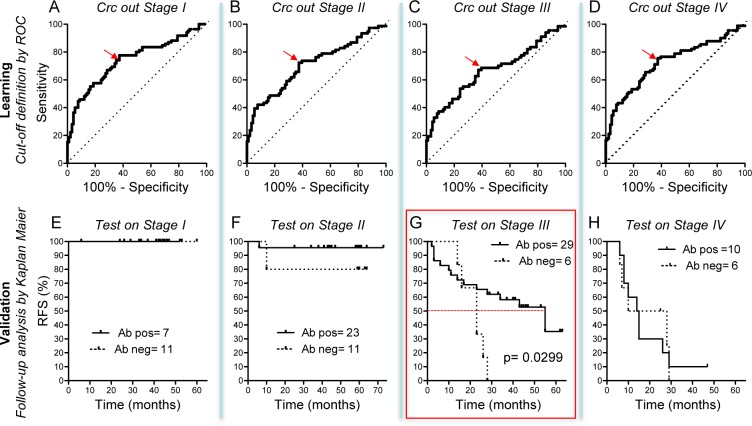
Patient follow up **A.**-**D.** Learning of the cut-off values for sera categorization (anti-ADAM10 Ab-positive or -negative) performed by ROC analysis of anti-ADAM10 optical density in Crc and Cn. In order to avoid over-fitting, the learning of the cut-off value (that was be applied to the group of patients in a specific stage of the disease) was done using all Crc patients excluding the group of patients at the stage that have been subsequently tested for the follow-up (Crc out Stage … = Crc without patients at specific stage). **E.**-**H.** Kaplan-Meier survival curve and Log-rank test analysis in the Crc patients at different stages. The analysis compares the patients showing immunoreactivity against ADAM10 (Ab-pos, solid line) *vs.* the patients with no reactivity (Ab-neg, dotted line). The considered outcome was recurrence-free survival (RFS) (patient alive, no relapse, no appearance of new metastasis), and the marked “events” were tumor relapse, metastasis and patient death. The dotted red line in panel G indicates the median RFS time significantly changing (from 23 to 55 months) in patients at stage III that showed immunoreactivity against ADAM10.

Since ADAM10 plays important physiological functions in epithelial cells, we investigate whether the anti-ADAM10 serological reactivity observed in Crc patients was a feature shared by other tumors of epithelial origin. The screening performed using patient sera from either epithelial tumors (pancreatic cancer, *n* = 43; breast cancer, *n* = 43) or hematopoietic malignancies (B-cell chronic lymphocytic leukemia, *n* = 53 and multiple myeloma, *n* = 46) showed a significant presence of auto-Abs against ADAM10 in pancreatic and breast cancer, but not in B-CLL and multiple myeloma (SM-Figure 2 and SM-Table 5), which in turn suggested that the reactivity against ADAM10 might be a marker of epithelial tumors.

### Auto-antibody response to ADAM10 is associated with favourable follow-up in stage-III disease patients

Since the anti-ADAM10 immunoreactivity was not present in all Crc patients, we investigated whether the occurrence of anti-ADAM10 auto-Abs was associated with the clinical outcome in patients at different disease stages. Therefore, we analysed the follow-up in a group of 97 Crc patients from both testing and validation cohorts, from which clinical outcome data was available. Follow-up was evaluated in a range from 2 to 73 months (mean 35±18.6 sd) considering recurrence-free survival (RFS) condition (patient alive, no relapse, no metastasis).

Sera reactivity for ADAM10 was categorized as positive or negative according to the threshold values defined by the receiver operating characteristic (ROC) curve analysis that give rise to the values of best trade-off between sensitivity and specificity. In order to avoid over-fitting, the learning of the cut-off value (that would be applied to the group of patients in a specific stage of the disease to determine their positive or negative ADAM10 reactivity) was done using all Crc patients excluding the group of patients at the stage that would be subsequently tested for the follow-up. For instance, the cut-off value learned excluding from the Crc cohort the Stage I group (Figure 2A, Crc out stage I) was subsequently tested for validation on the Stage I patients considering their follow-up (Figure 2E, Test on Stage I). The cut-off values learned for each of the four phases were close and the median cut-off value was 237.8 (Table 3, and Figure 2A-2D). Considering the learned cut-off, the validation based on Kaplan-Meier survival curve and Log-rank test indicated that the groups of patients at early stages (stage I and II), as well as the patients at advanced metastatic stage IV, did not show difference between patients that have or do not have anti-ADAM10 Abs. On the contrary, the follow-up of Crc patients with stage III of the disease showed that the presence of anti-ADAM10 Abs was associated with a significant prolonged RFS condition from 23 to 55 months (*p* < 0.0299; Hazard ratio HR = 0.23; 95% CI 0.06-0.86) (Table 4, and Figure 2E-2H). No other significant association were found between Crc patients’ demographic-clinical features (Table 1) and the presence or absence of anti ADAM10 auto-Abs. Interestingly, the presence of auto-Abs to ADAM10 and the disease stage were the only independent predictors of disease control (RFS; *p* = 0.041 and *p* < 0.0001, respectively) at multivariate Cox proportional hazards model analysis (Table 5).

**Table 3:** Definition by ROC analysis of the cut-off values for sera categorization

			OD Crc *vs*. Cn					
	Crc (n)	Cn (n)	(M-W)	ROC	AUC	cut-off (OD)	sens. (%)	spec. (%)
**Crc out Stg I**	85	91	*p* < 0.0001	*p* < 0.0001	0.74	264.9 (Stg I)	74.1	64.8
**Crc out Stg II**	76	91	*p* < 0.0001	*p* < 0.0001	0.70	237.2 (Stg II)	72.3	62.6
**Crc out Stg III**	67	91	*p* = 0.0004	*p* = 0.0003	0.66	238.4 (Stg III)	67.1	62.6
**Crc out Stg IV**	90	91	*p* < 0.0001	*p* < 0.0001	0.72	237.2 (Stg IV)	75.5	62.6

Crc= colorectal cancer; Cn= control subjects; Stg I-II-III-IV= tumour stage of colon-carcinoma patients; OD= optical density; M-W= Mann-Whitney test; ROC= receiver operating characteristic curve; AUC= area under the curve; sens.= sensitivity; spec.= specificity.

**Table 4:** Kaplan-Maier curve follow-up analysis for patients at different stages

				Sera (n)			RFS (months)			
	Crc (n)	cut-off (OD)	pos	neg	Log-Rank test	pos	neg	HR	95% CI
**Stg I**	18	264.9	7	11	*p* = 1	-	-	-	-
**Stg II**	28	237.2	23	5	*p* = 0.243	-	-	0.118	0.003-4.284
**Stg III**	35	238.4	29	6	*p* = 0.0299	55	23	0.232	0.062-0.867
**Stg IV**	16	237.2	10	6	*p* = 0.952	14.5	19	1.036	0.428-3.336

Crc= colorectal cancer; Cn= control subjects; Stg I-II-III-IV= tumour stage of colon-carcinoma patients; OD= optical density; pos= sera positive for anti-ADAM10 auto-antibodies; neg= sera negative for anti-ADAM10 auto-antibodies; RFS= recurrence-free survival; HR= hazard ratio; 95% CI= 95% confidence interval.

**Table 5:** Cox regression analysis of outcome predicting factor^°^

		multivariate		
		Reg. Coef.	HR (95% CI)	Chi^2^
Disease stage	III-IV *vs.* I-II	3.276	26.46 (6.24 - 112.16)	*p* < 0.0001
anti-ADAM10	pos *vs.* neg	−0.707	0.49 (0.24 − 1.01)	*p* = 0.041

° Recurrence-free survival; Reg. Coef.:= regression coefficient value; HR= hazards ratio; 95% CI= 95% confidence interval; pos vs. neg= anti-ADAM10 positive or negative sera

### ADAM10 protein is expressed in primary Crc tumor regardless of the presence of auto-Abs

**Figure 3:**
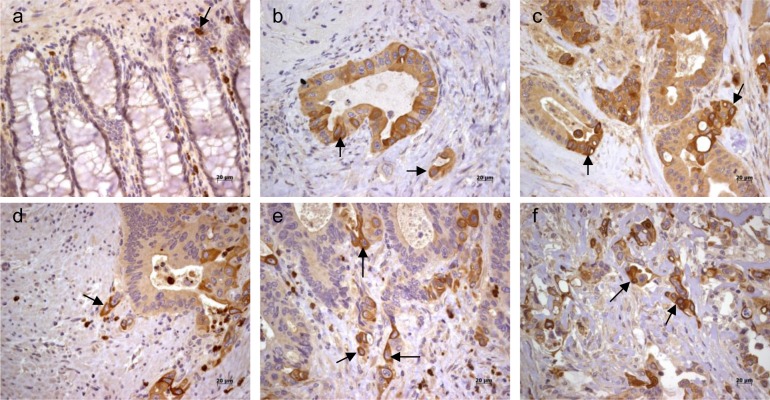
Expression of ADAM10 in primary tumor Immunohistochemistry analysis of ADAM10 expression in normal intestinal epithelia tissue **a.** and tumoral tissue **b.**-**f.**. Arrows indicated in panel **a.** the few ADAM10 positive monocytes resident in the cryptae, and in panels **a.**-**f.** the stronger reactivity of tumor cells invading the stroma.

The immunohistochemistry performed on 26 available tumor biopsies showed that ADAM10 was expressed at different levels in all primary tumors regardless of the presence of auto-Abs or different tumor stages (Figure 3 and SM-Table 6). Moreover, both the serological reactivity against ADAM10 and the tumor ADAM10 expression did not correlate with the degree of inflammatory lymphoid cells infiltrate found in the samples (SM-Table 6). Of interest was the pattern of reactivity of ADAM10 in the tumors. While the normal control intestinal epithelia did not express ADAM10, with the exception of a reactivity observed in scanty infiltrating cells (Figure 3a, indicated by the arrow), the transformed epithelia showed heterogeneity in the intensity of the ADAM10 expression (panels b and c). ADAM10 was highly expressed in the tumoral cells that seem to move out from the epithelia organization (arrows in panels d and e) and in the budding area of the tumor invading the stroma (arrows in panels e and f).

### Immature ADAM10 is highly expressed in Crc tumors of anti-ADAM10-positive patients

**Figure 4:**
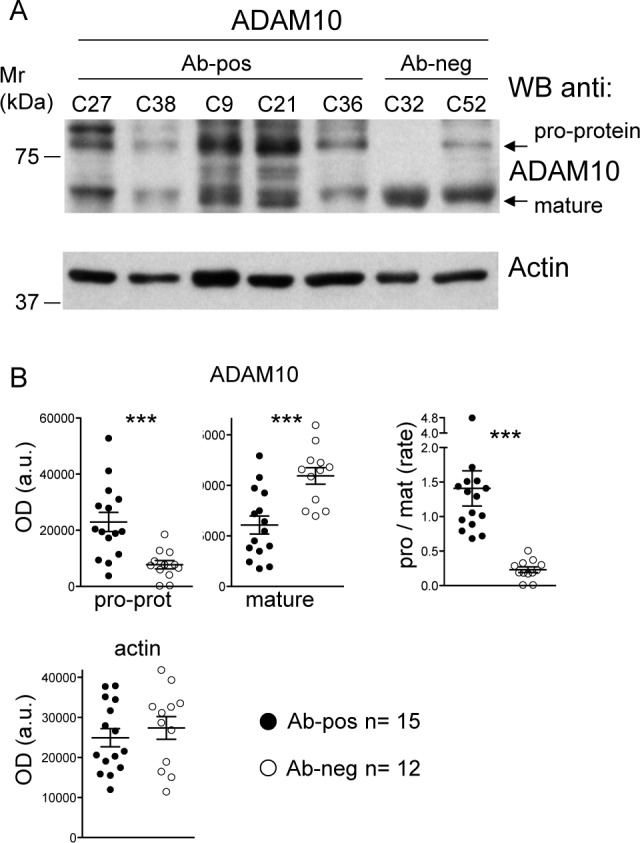
ADAM10 isoforms expression in Crc specimens **A.** Anti-ADAM10 WB analysis in Crc specimens from representative patients with (Ab-pos, C27, C38, C9, C21, C36) or without (Ab-neg, C32, C52) serological reactivity against ADAM10. Anti-β-actin reactivity was used for protein-loading control and OD normalization. **B.** Quantitative analysis of WB reactivity detected on Ab-pos (*n* = 15) and Ab-neg (*n* = 12) patient specimens (mean +/− SEM 3 experiments; student's *t*-test; ** *p* < 0.01; *** *p* < 0.001). The ratio between pro-protein and mature ADAM10 signals is reported.

The expression of different ADAM10 isoforms, namely the 64 kDa mature/active isoform and the 98 kDa immature/inactive pro-protein, has been evaluated by WB on the available tumor tissues from Crc patients. The specimens included patients with positive (Ab-pos, *n* = 15) and negative (Ab-neg, *n* = 12) serological reactivity against ADAM10 (SM-Table 1 and 2). ADAM10-Ab-pos patients showed higher expression of the immature pro-protein (*p* = 0.0009 student's *t*-test), and lower expression of the mature ADAM10 (*p* = 0.0006) than ADAM10-Ab-neg patients, which in turn resulted in the latter in a reduced pro-protein/mature ADAM10 ratio (*p* = 0.0004) (Figure 4A and 4B).

### Anti-ADAM10 auto-Abs from patient sera recognize the immature ADAM10

**Figure 5:**
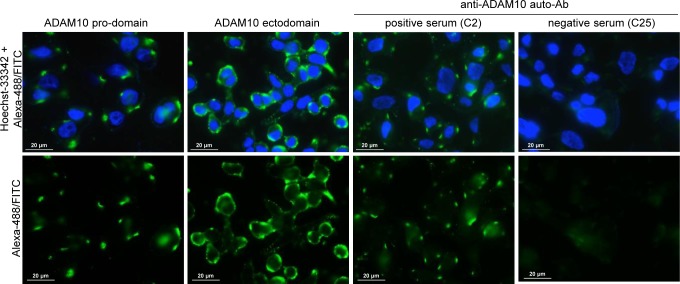
Immunofluorescence reactivity of IgG from patient sera on LoVo cell line ADAM10 pro-domain: reactivity of the rabbit polyclonal anti-ADAM10 pro-domain-specific Ab (ab39178, Abcam), which recognized the immature non-functional isoform of ADAM10, showed patched reactivity. ADAM10 ectodomain: reactivity of the goat polyclonal anti-ADAM10 ectodomain-specific Ab (R&D-Systems, AB936), which recognized both the immature and mature (cleaved and functional) isoforms of ADAM10, showed patched and diffuse signals. Anti-ADAM10 auto-Ab positive serum: reactivity of the human IgG fraction purified from a representative serum (C2) of Crc patients considered positive for the presence of auto-Ab anti ADAM10, showed patched reactivity. Anti-ADAM10 auto-Ab negative serum: no reactivity was observed for the human IgG fraction purified from serum (C25) of a representative Crc patient considered negative for the presence of auto-Ab anti ADAM10. Cell nuclei were stained with Hoechst-33342. The reactivity of the secondary antibodies (goat anti-rabbit IgG Alexa-488; donkey anti-goat IgG Alexa-488 and goat anti-human IgG FITC) are shown as negative controls in SM-Figure 3. Images were acquired by immunofluorescence microscopy (Zeiss Upright Axo Imager 2 equipped with AxoVision Rel.4.8.2 software); magnification 63X. Images were linearly adjusted for brightness and contrast using Adobe-Photoshop CS4 v.11 software.

The purified IgG fraction from sera of representative Crc patients either considered positive (*n* = 6; C27, C2, C16, C20, C14, C19) or negative (*n* = 6; C11, C32, C12, C25, clcr14, C13) for anti-ADAM10 reactivity (SM-Figure 3) was used for an immunofluorescence assay performed on the LoVo cell line. These cells were chosen based on the high expression level of immature ADAM10, due to a deficient activity of furin pro-protein convertase which results in an unusual cell surface expression of the immature ADAM10 [20, 21]. The reactivity of the anti-ADAM10-pro-domain and anti-ADAM10-ectodomain Abs confirmed that LoVo cells did express both immature and mature ADAM10 on the cell surface (Figure 5). Interestingly, the prevalent reactivity pattern of the pro-domain-specific Ab showed a few bright large aggregates/patches (Figure 5), while the reactivity of the Ab specific for the ADAM10-ectodomain, in addition to the bright aggregated signals, showed a diffuse signal likely corresponding to the surface distribution of the mature ADAM10 (Figure 5). Thus, the bright/patched signals correspond to the distribution of the immature isoform of ADAM10 that seems to form clusters of molecules on the cell surface.

**Figure 6:**
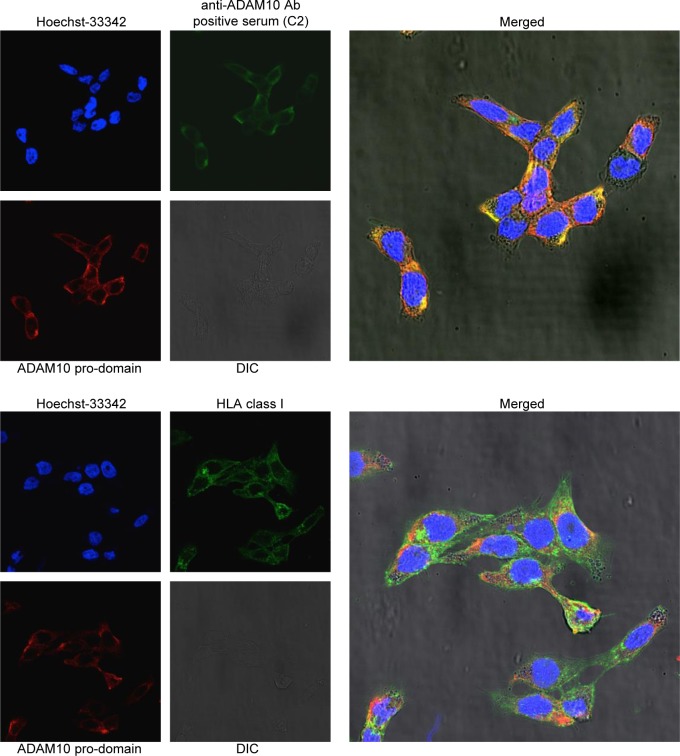
Co-localization of immunofluorescence signals from anti-ADAM10 pro-domain Ab and IgG from anti-ADAM10 positive patient serum Double staining was performed with rabbit anti-ADAM10 pro-domain (ab39178, Abcam), and either IgGs purified from Crc patient serum or mouse anti-HLA class I (Santa-Cruz Biotechnology). Secondary Abs were Alexa-546-conjugated goat-anti-rabbit IgG and Alexa-488-conjugated rabbit-anti-mouse IgG or FITC-conjugated goat-anti-human IgG. Cell nuclei were stained with Hoechst-33342, and differential interference contrast (DIC) images were also acquired. Staining was assessed by immunofluorescence confocal microscopy (Leica TCS SP5 Laser Scanning Confocal) and images acquired with LAS-AF (Leica) software; magnification 63X. Images from single channel and merged images are shown. Images were linearly adjusted for brightness and contrast using ImageJ 1.47v software.

Of note, the reactivity of IgG from patients positive for anti-ADAM10 auto-Abs was similar to the patched signal of immature ADAM10 (Figure 5 and SM-Figure 4), or in one case, in addition to patched signals, a diffuse reactivity was evidenced (SM-Figure 4-C27). Conversely, IgG from patients negative for anti-ADAM10 auto-Abs did not show reactivity at all (Figure 5 and SM-Figure 4), except one case that showed a weak homogeneous reactivity with surface membrane pattern (SM-Figure 4-clcr14). The double-staining performed with the anti-ADAM10-pro-domain Ab and with the IgGs purified from patients positive for anti-ADAM10 auto-Abs showed a co-localization of the patched signals, suggesting that patient auto-Abs also recognize the immature ADAM10 isoform (Figure 6). On the contrary, no signal co-localization was observed if the double staining was performed with the anti-ADAM10 pro-domain Ab and the anti-HLA class I Ab, as control (Figure 6).

**Figure 7:**
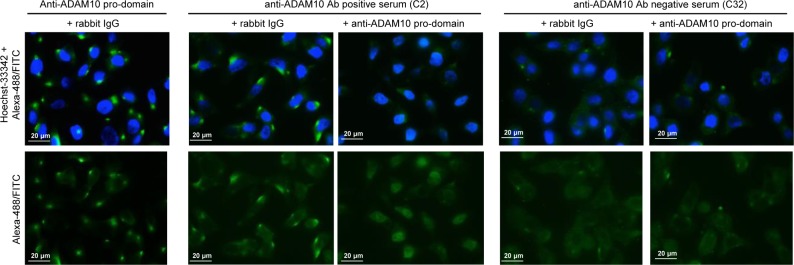
Immunofluorescence reactivity after competition with anti ADAM10 pro-domain Ab Anti-ADAM10 pro-domain: reactivity of the rabbit anti-ADAM10 pro-domain Ab (ab39178, Abcam) after competition with control rabbit IgG. Anti-ADAM10 positive serum (C2): reactivity of the IgG fraction purified from a representative serum of a Crc patient considered positive for the presence of auto-Abs anti ADAM10 after competition either with control rabbit IgGs or the anti-ADAM10 pro-domain Ab. Anti-ADAM10 negative serum (C32): reactivity of the IgG fraction purified from a representative serum of a Crc patient considered negative for the presence of anti ADAM10 auto-Ab after competition either with control rabbit IgGs or anti-ADAM10 pro-domain Ab. Cell nuclei were stained with Hoechst-33342; secondary Abs were goat anti-rabbit IgG Alexa-488 and goat anti-human IgG FITC. Images were acquired by immunofluorescence microscopy (Zeiss Upright Axo Imager 2 equipped with AxoVision Rel.4.8.2 software); magnification 63X. Images were linearly adjusted for brightness and contrast using Adobe-Photoshop CS4 v.11 software.

The recognition of the same target was inferred by competition experiments, and indeed the patched reactivity of IgG from patients positive for anti-ADAM10 auto-Abs was inhibited by competition with the anti-ADAM10-pro-domain Ab, but not by competition with control rabbit IgG (Figure 7 and SM-Figure 5). In addition, competition with the anti-ADAM10-pro-domain Ab did not affect the reactivity of the IgGs purified from patients considered negative for anti-ADAM10 Abs even in the case of the patient showing weak diffuse reactivity (Figure 7 and SM-Figure 5). Collectively, these results, the staining pattern similarity, the signal co-localization and the competition experiments, indicated that the target recognized by patient auto-Abs was the ADAM10 pro-domain.

### Pro-domain of ADAM10 is the target of the anti-ADAM10 auto-Abs from Crc patients

In order to confirm the specificity of the auto-Abs from Crc patients toward the ADAM10 pro-domain we used the purified IgG fraction of representative Crc patients, either considered positive or negative for anti-ADAM10 reactivity (positive *n* = 6; C2, C27, C20, C9, C14, C19; negative *n* = 6; C32, C50, C25, C11, C13, C45), in a dot-blot analysis against recombinant ADAM10 molecules either containing or not the pro-domain, namely immature and mature ADAM10. Reactivity was also tested against a synthetic peptide of 32 aa sequence within the pro-domain.

**Figure 8:**
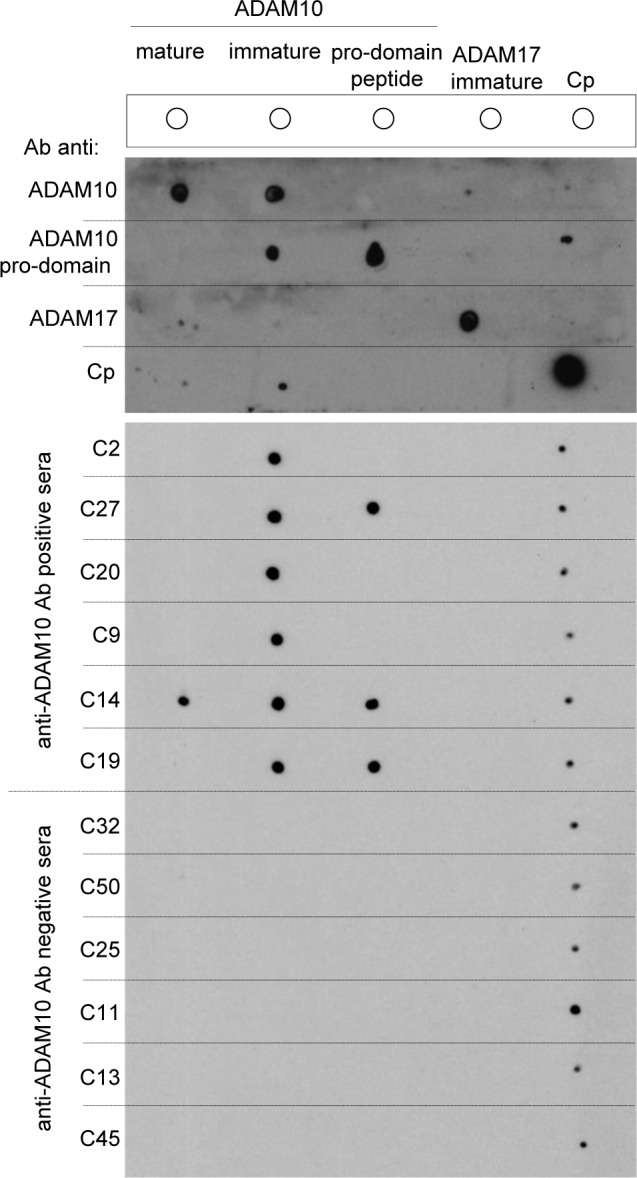
Dot blot reactivity of IgG from patient sera on recombinant ADAM10 molecules The reactivity of the IgG fraction purified from the sera of 6 Crc patients positive for the presence of auto-Abs anti-ADAM10 (C2, C27, C20, C9, C14, C19) and 6 Crc patients considered negative (C32, C50, C25, C11, C13, C45) was tested on human-recombinant ADAM10 (mature form lacking the pro-domain), mouse-recombinant ADAM10 (immature form including the pro-domain), ADAM10 pro-domain synthetic peptide (32 aa within the pro-domain sequence), human-recombinant ADAM17 (immature form including the pro-domain) and purified human ceruloplasmin (Cp) proteins spotted (200 ng/spot) onto nitrocellulose membrane. The reactivity of the anti-ADAM10 ectodomain Ab that recognizes both mature and immature ADAM10, anti-ADAM10 pro-domain Ab, anti-ADAM17 and anti-ceruloplasmin Abs were used as controls.

The results showed that the IgG from Crc patients positive for anti-ADAM10 reactivity specifically recognize the pro-domain being reactive with mouse-recombinant immature ADAM10 (aa 19-673) and not reactive with the human-recombinant mature ADAM10 (aa 214-672), which does and does not contain the pro-domain, respectively (Figure 8). In addition some of these sera (C27, C14, C19) also reacted with the pro-domain synthetic peptide, that likely was part of the recognized epitope/s. The ADAM10-positive serum C14, in addition to pro-domain recognition inferred by the reactivity against the pro-domain peptide, also showed a signal against the ectodomain of ADAM10, which in turn suggested the presence of a polyclonal reactivity (Figure 8). On the contrary to the anti-ADAM10 positive sera, the IgG from patients considered negative for anti-ADAM10 reactivity did not show reactivity with recombinant ADAM10 molecules nor with the ADAM10 pro-domain peptide (Figure 8).

A weak cross-reactivity toward the human ceruloplasmin purified from plasma was detectable for all of the IgG purified from patients, while no cross-reactivity was observed with ADAM17. These two molecules were used as control for non-specific binding toward molecules related or not-related to ADAM10, ADAM17 and ceruloplasmin, respectively. Of note, the purified recombinant human ADAM17 (aa 1-671) contains its own pro-domain and a His-Tag as well as the mouse recombinant ADAM10, so we were able to rule out any reactivity against the tag and to confirm the specificity for ADAM10 pro-domain. Moreover, the dot blot results showed that the commercial anti-ADAM10 antibodies properly recognized the ADAM10 ectodomain or pro-domain accordingly to their own specificity.

In conclusion, the immunofluorescence results together with the dot-blot analysis clearly shows the specificity for the ADAM10 pro-domain of the patient's auto-antibody.

## DISCUSSION

The first inferrence from our work is that by applying the SERPA approach to the membrane protein enriched material it is possible to highlight specific immunoreactivities that provide candidates for both companion diagnostic/prognostic markers and surface antigens that might serve as therapeutic targets. Exploiting this approach, we identified the surface membrane enzyme ADAM10 as a candidate antigen able to elicit a humoral immune response in colon cancer. ADAM10 is characterized by a multi-domain organization that includes inter alia a metalloproteinase domain and a N-terminus pro-domain [22, 23]. ADAM10 is synthesized as an inactive zymogen of 98 kDa that is processed to the mature form of 64 kDa in the trans-Golgi compartment where the pro-domain is removed by a proprotein convertase such as PC7 or furin [24]. The mature ADAM10 is then transported to the plasma membrane in association with different members of the tetraspanins protein family that can regulate its transport and functions [25–28]. On the plasma membrane ADAM10 can act in shedding the ectodomains of several different membrane-bound substrates which include the precursor forms of growth factors, cytokines, growth- and cytokine-receptors, and various adhesion molecules (reviewed in [22, 23, 29, 30]); therefore, its dysregulation has been reported to have a role in cancer progression and invasion [31–35]. In colon cancer, overexpression of ADAM10 has been reported to correlate with an advanced stage of disease and with enhanced induction of metastasis [36, 37]. For these reasons, ADAM10 has been proposed as a target for anti-cancer therapy [29, 38, 39], in particular its functional inhibition might be efficacious in reducing activation of EGF-receptors [32, 40].

The induction of a serological immune response against ADAM10 that we found in Crc patient sera underline the important role that this metalloprotease has in the epithelial cancer physio/pathology. The presence of auto-Abs to ADAM10, *per* se, cannot be considered as a disease biomarker; while a considerable fraction of patients (74%) displayed this reactivity, there is an absence of a relevant specificity (62%). The auto-Abs to ADAM10 might rather reflect a peculiar molecular asset of the tumor in this group of patients, which in turn results in a limitation of the disease progression and a more favorable outcome. Indeed, anti-ADAM10 auto-Abs were associated with an impressive prolonged recurrence-free survival condition in the patients at stage III of the pathology when lymph nodes start to be infiltrated by the tumor cells. This is a critical step of the disease where the humoral response against ADAM10, or a reduced ADAM10 functionality, might confer an advantage in limiting the progression of the tumor. Therefore the presence of anti-ADAM10 Abs in patient sera can be considered as a favourable feature in patients with Crc at stage-III that undergo surgical resection of the tumor. This is a clinically relevant feature because, in addition to the disease stage, the presence of anti-ADAM10 auto-Abs may be helpful to better stratify the stage III patients providing a more reliable clinical outcome prediction.

The mechanism involved in the production of auto-Abs to ADAM10 in Crc patients is presently unclear. It is known that specific immunogenic epitopes may be generated during tumoral transformation by aberrant post-translational modifications or by the expression of different isoforms of specific proteins [1, 10, 15]. In our study, the unusual abundance of immature ADAM10 isoform expressed in the tumor of auto-Ab-positive patients seems to be responsible for the immunogenicity as suggested by the reactivity of patients’ IgG against the ADAM10 non-cleaved pro-domain. Under physiological conditions, the ADAM10 zymogen shows an intracellular localization [23], however, in the absence of a correct pro-convertase activity a surface membrane expression of the immature ADAM10 has been reported in the LoVo cell line [20, 21, 41], as confirmed by the reactivity of the anti-prodomain Ab we found in the same cells. Therefore, similarly to LoVo cells, the increased expression of immature ADAM10 in the tumor of patients with anti-ADAM10 serological reactivity might be due to a reduced/aberrant processing of ADAM10, for example as the result of a defective expression/function of the proprotein convertases necessary for the pro-domain removal. Other possible mechanisms may include an altered expression of the tetraspanin proteins described to interact with the transmembrane domain of immature ADAM10 and to regulate its maturation and trafficking from the endoplasmic reticulum to the surface membrane [25–28, 42], thus a tetraspanin aberrant expression could prevent ADAM10 pro-domain cleavage.

In the present study, the analysis of the immature ADAM10 on the patient tumor sections was not possible, since in our hands the currently available anti-ADAM10 pro-domain reagent did not work in immunohistochemistry; therefore it is yet to be assessed whether, in addition to an increased expression, the immature ADAM10 is also expressed on the surface of the tumoral cells in patient tissue.

Cancer progression is often associated with the ability to escape immune responses, thus, the association of anti-ADAM10-pro-domain auto-Abs with the favorable clinical outcome of patients at stage III might reflect a more efficient immune response in these patients. In this context, it would be of interest to investigate whether the induction of auto-Abs to immature ADAM10 might reflect the activation of specific CD4+T helper cells able to foster an active anti-tumor response. Alternatively, the detected immunoreactivity might simply be a read-out elicited by the aberrant surface expression of the immature non-functional ADAM10 isoform, and the explanation for the observed favorable clinical outcome can be related to an alteration of the ADAM10 functions that are associated with tumor progression and invasion [31–37]. In accordance with a reduced/aberrant processing and an increased expression of immature ADM10, the overall sheddase activity of ADAM10 could be reduced on the membrane of the tumor cells of the anti-ADAM10-positive patients, which in turn would result in an impairment of tumor growth and invasion. Since the auto-Abs raised in the patients specifically reacted with the ADAM10 pro-domain, it is unlikely that they act in inhibiting the ADAM10-sheddase activity *in vivo* and are contributing to tumor progression constraint.

In conclusion, the presence of anti-ADAM10 auto-Abs is a feature associated with favorable prognosis for stage-III Crc patients that show increased tumoral expression of the immunogenic immature/inactive ADAM10. In these patients, the reduced ADAM10 maturation might result in a decrease of sheddase activity that could reduce the proliferative and invasive capacity of tumor cells. In the future, it will be of interest to investigate the Crc expression levels of proprotein convertases and teraspanins as molecular characteristics of the tumor that might correlate with both the presence of anti-ADAM10 auto-Abs and the favourable prognosis.

## MATERIALS AND METHODS

### Sera and tissue samples

Samples were obtained following written informed patient consent and approval by the Institutional Ethical Committee (SCCD4 01/03 and DO/MS/ER/mm n. 071/11); the study has been conducted according the principles expressed in the Helsinki Declaration. As testing cohorts we used the sera collected in our previous study from patients with Crc (*n* = 57), and from the control group (*n* = 39) that included healthy subjects and patients with non-tumoral pathologies to exclude reactivity associated with general pathological condition [15]. The Crc patients with different disease stages were further considered as sub-groups (stage I, *n* = 8; stage II, *n* = 17; stage III, *n* = 26; stage IV, *n* = 6). Validation cohorts included sera collected throughout the span of two years, from Crc patients (*n* = 49) at the time of surgical resection, and from healthy subjects (*n* = 52). Also in the Crc validation cohort, patients with different stages were considered as sub-groups (stage I, *n* = 13; stage II, *n* = 13; stage III, *n* = 13; stage IV, *n* = 10). Sera were isolated from venous blood, aliquoted and stored at −80°C until use. De-identified numeric specimen codes were used to protect the identity of the individuals. Diagnosis of Crc was assessed by histological or cytological analysis performed by the Pathology Unit, San Raffaele Hospital. Age and sex distribution resulted to be homogeneous in testing and validation cohorts, both intra- and inter-cohorts (Table 1; for single subjects’ features see SM-Table 1-4). Tumor specimens available for the study were obtained from the Pathology Unit, San Raffaele Hospital, and were from the resected colon either frozen at −80°C or embedded in paraffin and stored at −20°C according to the hospital Pathology Unit procedures. Patient follow-up was carried out through chart review and patient interviews by the surgical team who was blinded to the experimental results. The follow-up analysis was done from the time of surgical resection considering recurrence-free survival (RFS) condition (patient alive, no relapse, no appearance of new metastasis); the recorded events were tumor relapse, metastasis occurrence and patient death. If none of these events occurred, survival was censored at the time of the last visit.

### Cell lines

LS180 primary colon adenocarcinoma and LoVo metastatic colon carcinoma cell lines (American Type Culture Collection) were used. The cells were grown under standard conditions, used at early passages and medium was tested monthly by PCR for mycoplasma contamination.

### Membrane proteins biotinylation and purification

LS180 cells were surface biotinylated and membrane proteins were purified on ImmunoPure immobilized monomeric avidin columns (Pierce) as previously described [43]. Antibodies (Abs) specific for surface membrane proteins (CD46 and ß1-integrin, Abcam), and intracellular proteins (ß-actin and ß-tubulin, Sigma) were used in WB to check the quality of purification.

### 2DE, WB and image analysis

2DE of biotinylated material was performed as reported in [15], proteins were transferred to nitrocellulose membrane and incubated for 12 hr at 4°C either with discovery Crc patient pooled sera or with control subject pooled sera (1:300 dilution), both from our previous study [15]. Immunodetection was revealed with HRP-conjugated anti-human-IgG (Southern Biotechnology) followed by ECL reaction and film exposure. Preparative 2DE was stained with Coomassie blue; gels or films were acquired with Laser-Densitometer (Molecular Dynamics), and spot patterns investigated using Progenesis-PG240 software (Nonlinear Dynamics).

### Serological screening on purified ADAM10

Recombinant extracellular domain of mouse ADAM10 (amino acids 19-673 including pro-domain; 97% homology with human) (Calbiochem) was resolved (200 ng/lane) by SDS-PAGE, and transferred to nitrocellulose membrane. Membrane strips were incubated with patient or control sera (50 μg/ml of IgG working dilution) and reactivity revealed as described above. The optical density (OD) of sera reactivity was evaluated using ImageQuant software (GE-Healthcare). Each serum was tested twice and, after background subtraction, OD values were normalized to the reactivity of the anti-ADAM10 ectodomain Ab (AB936, R&D Systems) obtained in the same film exposure.

### Statistical analysis

Statistical analyses were performed using GraphPad Prism V5 or *X*LSTAT softwares, in all analyses a two-sided *p* value < 0.05 was considered to be significant. Categorical data were analysed using Fisher's test and two-tailed *p* value, including the association between clincal-pathological variables and the presence of anti-ADAM10 autoantibodies. Continuous data were evaluated using unpaired student's *t*-test with two-tailed p value for the comparison of two means with standard deviation or standard error, if data-sets showed normal distribution as inferred by Kolmogorov-Smirnov test, or by Mann Whitney test in the case of non-Gaussian distribution. Nonparametric analysis of variance, including post-analysis, was performed using Kruskal-Wallis test. Correlation analysis was done using Spearman's rank coefficient. The receiver operating characteristic (ROC) curve analysis was used to define the threshold value for OD that gave the better ratio between sensitivity and specificity and that was than used to consider a serum positive or negative for the presence of auto-Ab. In order to avoid over-fitting, the learning of the cut-off value (that would be applied to the group of patients in a specific stage of the disease to determine their positive or negative ADAM10 reactivity) was done using all Crc patients excluding the group of patients at the stage that would be subsequently tested for the follow-up. The patients' follow-up as recurrence-free survival time was evaluated by Kaplan-Meier survival curve and Log-rank test analysis. The Hazard Ratio (HR) and confidence limits at 95% were estimated for each variable using Cox univariate model. A multivariate Cox proportional Hazard model was also developed using stepwise regression (forward selection) with predictive variables that were significant at univariate analysis. The cut-off P-values to enter in or to be removed from the multivariate model were set at 0.1 and 0.3, respectively.

### Mass spectrometry analysis

Protein spots excised from gel were digested with trypsin (Roche Diagnostics), and peptides were analysed on an API-QStar-PULSAR (AB-Sciex) mass spectrometer searching in the UniProt Human Complete Proteome cp hum 2012 11 database as previously described [44]. Protein identity was confirmed on LS180-biotinylated material by 2DE-WB using an anti-ADAM10 Ab (AB936, R&D Systems).

### Immunofluorescence analysis

Cell were fixed with 4% paraformaldehyde and either permeabilized with 0.5% saponin or used as they were for immunostaining with goat anti-ADAM10 (AB936, R&D-Systems), rabbit anti-ADAM10 pro-domain (ab39178, Abcam), mouse anti-HLA class I (Santa-Cruz Biotechnology), mouse anti-ß-actin (Sigma) and IgGs purified from Crc patient sera (50 μg/ml). Alexa-488-conjugated goat-anti-rabbit IgG, donkey-anti-goat IgG and rabbit-anti-mouse IgG (Invitrogen) or FITC-conjugated goat-anti-human IgG (Southern Biotechnology) were used as secondary Abs; in double staining experiments the Alexa-546-conjugated goat-anti-rabbit IgG (Invitrogen) was used. Staining was assessed either by immunofluorescence microscopy (Zeiss Upright Axo Imager 2) or confocal microscopy (Leica TCS SP5 Laser Scanning Confocal), and images acquired with AxoVision Rel.4.8.2 (Zeiss) or LAS-AF (Leica) software, respectively. Binding-competition was performed by pre-incubating cells (7 hr at 4°C) with 50 μg/ml of either rabbit-anti-ADAM10 pro-domain (ab39178, Abcam) or purified rabbit IgG before immunofluorescence staining with IgG purified from patient sera.

### ADAM10 protein expression in Crc by immunohistochemistry and western blot (WB)

Formalin-fixed, paraffin-embedded sections from available Crc specimens and normal mucosa underwent antigen retrieval and quenching with 3% hydrogen peroxide. Sections were incubated 12 hr at 4°C with anti-ADAM10 N-terminal antibodies (1:500 Sigma; 1:1000 ab39153, Abcam), followed by labelled Envision anti-rabbit System (Dako). The immunoreaction was revealed by HRP using 3,3′diaminobenzidine (Biogenex), the slides were slightly counterstained with Harris's hematoxylin, analysed with optical microscope Axioskope 2 (Zeiss) and images acquired with AxioVision 4.4 system (Zeiss). Available tumor specimens frozen at −80°C were mechanically broken and incubated with 1%TritonX-extraction buffer containing protease- and phosphatase-inhibitors (Sigma). Proteins were resolved by SDS-PAGE and WB analysis was performed with anti-ADAM10 (AB936, R&D-Systems) and anti-β-actin (Sigma) Abs. Secondary Abs were HRP-conjugated goat-anti-rabbit IgG and rabbit-anti-mouse IgG (Dako). The protein loading was normalized by ponceau-staining and β-actin immunostaining.

### IgG purification from Crc patient sera

Crc patient sera (250 μl each, 1:4 in PBS) were mixed with Protein-G-Agarose (Invitrogen) (200 μl dry beads) and incubated 24 hr at 4°C. Bound IgGs were eluted with 0.1 M Glycine-HCl (pH 2.6) and immediately buffered adding 1M Tris, then were concentrated at 50 mg/ml in PBS.

### Dot blot analysis on recombinant ADAM10 molecules

Human-recombinant ADAM10 (aa 214-672, lacking the pro-domain, R&D Systems 936-AD-020), mouse-recombinant ADAM10 (aa 19-673, including the pro-domain, Calbiochem PF124), ADAM10 pro-domain synthetic peptide (Abcam ab41165; 32 aa within the pro-domain, sequence not available because of property used as immunogen to generate the anti-ADAM10 pro-domain antibody), human-recombinant ADAM17 (aa 1-671, including the pro-domain, Calbiochem PF133) and human ceruloplasmin purified from plasma (Alexis Biochemicals, ALX-200-089) were spotted (200 ng each spot) onto nitrocellulose membrane and tested with the fraction of IgG purified from the sera of six Crc patients considered positive for the presence of auto-antibodies anti-ADAM10 and six patients considered negative. Membrane strips were incubated for 2 hr at 20°C with IgG purified from Crc patients sera (50 μg/ml of IgG working dilution) and reactivity revealed with HRP-conjugated anti-human-IgG (Southern Biotechnology) followed by ECL reaction and film exposure. As controls, the reactivity of the rabbit anti-ADAM10 Ab (Abcam, ab39153) that recognizes both mature and immature ADAM10, rabbit anti-ADAM10 pro-domain Ab (Abcam, ab39178), rabbit anti-ADAM17 (Abcam, ab39163) and sheep anti-ceruloplasmin (Abcam, ab8813) were tested on the spotted proteins. Immunoreactivities were revealed as described above.

## SUPPLEMENTAL MATERIAL



## Abbreviations

2DE: two-dimensional electrophoresis
ADAM10: A Disintegrin And Metalloproteinase 10
Abs: antibodies
Cn: control subjects
Crc: colorectal cancer
ECD: extracellular domain
MS: mass spectrometry
OD: optical density
SERPA: serological proteome analysis
WB: western blot.
