# Supplementary material for: Serological immune response against ADAM10 pro-domain is associated with favourable prognosis in stage III colorectal cancer patients
Source: Oncotarget. 2016 Aug 10;7(48):80059–76. doi: 10.18632/oncotarget.11181 (PMC5346771; doi:10.18632/oncotarget.11181)
Supplement: Supplementary file 1 [file oncotarget-07-80059-s001.pdf]

Serological immune response against ADAM10 pro-domain is associated with favourable prognosis in stage III colorectal cancer patients

Supplemental Material

Supplemental Figure 1-5

Supplemental Table 1-6

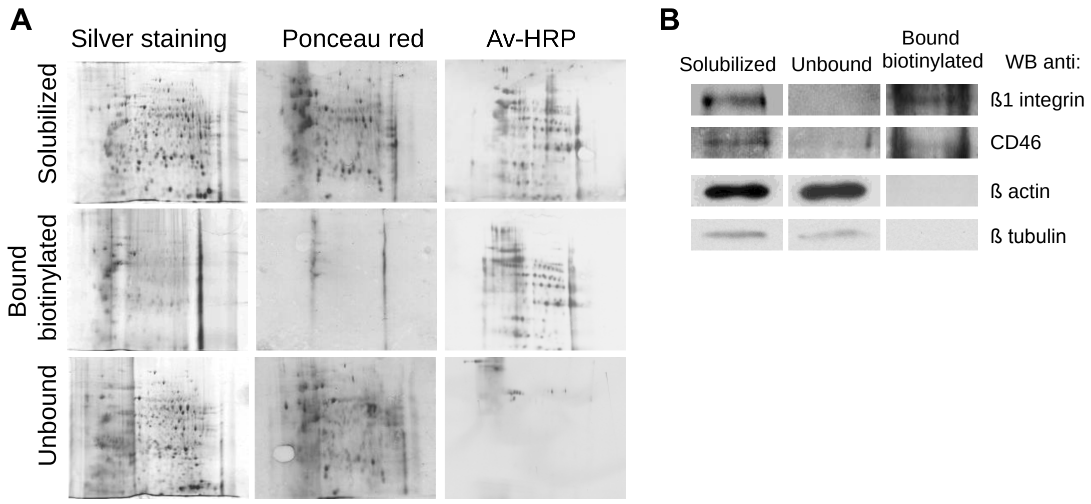

Supplemental Materials SM-Figure 1. *Enrichment of colon cancer cell line surface membrane proteins.* A) Enrichment by affinity chromatography of surface biotinylated proteins of LS180 colon cancer cell line resolved by 2D-electrophoresis; silver staining and ponceau red detection of protein showed a great number of spots in Total lysate and the fraction of material Unbound to the column, and few spots in Bound material; the latest was strongly reactive with streptavidin-HRP. B) WB analysis of membrane protein enrichment. Total lysate, material unbound to column and biotinylated material bound to column were resolved by SDS-PAGE and challenged with Abs specific form membrane protein (ß1-integrin and CD46) and cytoskeletal proteins (ß-actin and ß-tubulin).

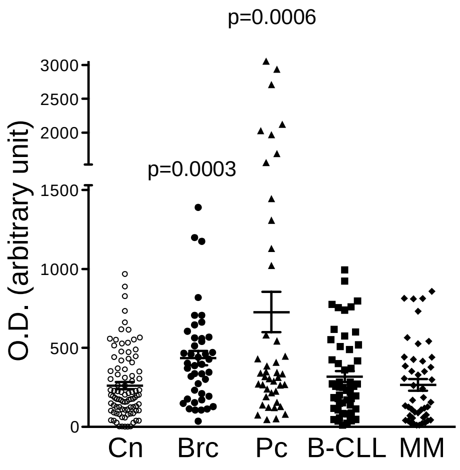

Supplemental Materials SM-Figure 2. S*erological reactivity against purified ADAM10 of sera from patients with tumors of epithelial and non-epithelial origin*. Quantitative analysis of serological reactivity was obtained, as for Crc sera, by western blot against purified ADAM10 using sera from cohorts of patients with tumors of epithelial and non-epithelial origin. Values are reported as optical density (mean +/- SEM of 3 experiments in duplicate) normalized to the anti-ADAM10 Ab reactivity. Statistical analysis was performed by Mann-Whitney test. Cn, control subjects n=91; Pc, pancreas carcinoma n= 43; Brc, breast cancer n= 43, MM, multiple myeloma n= 46; B-CLL, B-cell chronic lymphocytic leukemia n= 53.

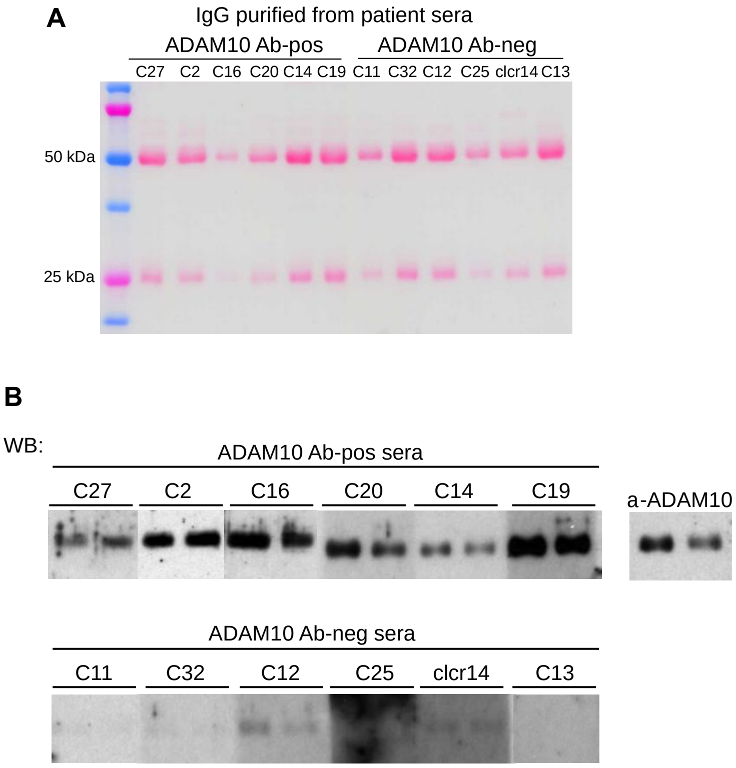

Supplemental Materials SM-Figure 3. *Purification of the IgG fraction from Crc patients sera.* A) IgG purified from Crc patient sera. Five µl of purified IgG from Crc patients with positive (ADAM10 Ab-pos) (C27, C2, C16, C20, C14, C19) or negative (ADAM10 Ab-neg) (C11, C32, C12, C25, clcr14, C13) anti-ADAM10 serological reactivity were resolved by SDS-PAGE under reducing conditions. Ponceau red staining showed heavy (50 kDa) and light (25 kDa) chains of the IgGs. B) Representative immunoreactivity of the IgGs purified from Crc patient sera on recombinant ADAM10 (in duplicate) showed the retention of the specific serologic reactivity for purified IgGs as recorded for the sera *in toto* from the same patients.

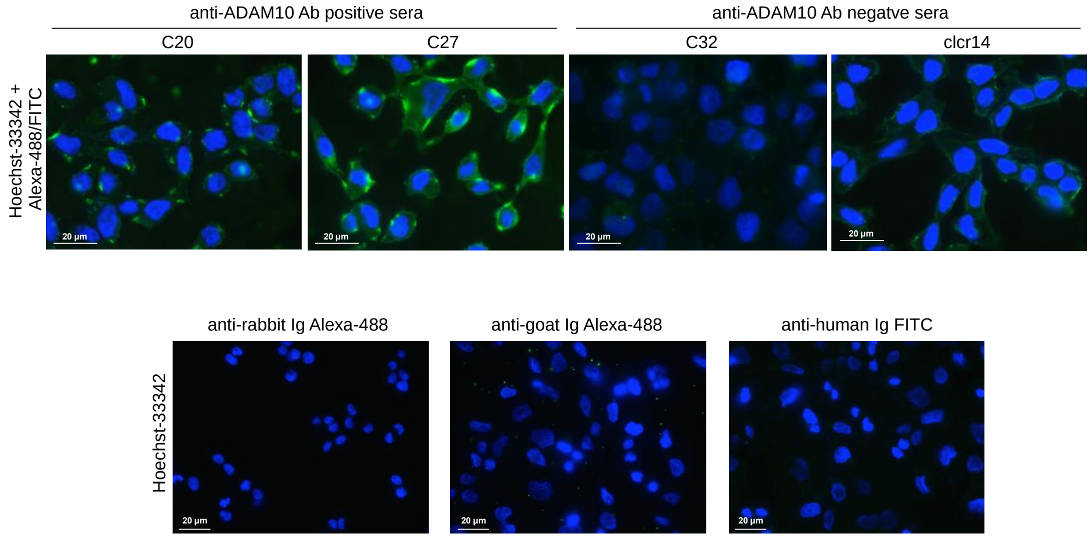

Supplemental Materials SM-Figure 4. *Immunofluorescence reactivity of IgG from patient sera on LoVo cell line.* Anti-ADAM10 Ab positive sera: reactivity of the human IgG fraction purified from representative serum of Crc patients (C20 and C27) considered positive for the presence of anti ADAM10 auto-Abs. For serum C27, in addition to patched signals, a diffuse reactivity was evidenced. Anti-ADAM10 Ab negative sera: no reactivity was observed for the human IgG fraction purified from the serum of representative Crc patient (C32) considered negative for the presence of anti ADAM10 auto-Ab. In one case of patients negative for anti-ADAM10 auto-Ab (clcr14) a weak homogeneous reactivity with surface membrane pattern was observed. Cell nuclei were stained with Hoechst-33342. The reactivity of the secondary antibodies (goat anti-rabbit IgG Alexa-488; donkey anti-goat IgG Alexa-488 and goat anti-human IgG FITC) are shown as negative controls. Images were acquired by immunofluorescence microscopy (Zeiss Upright Axo Imager 2 equipped with AxoVision Rel.4.8.2 software); magnification 63X. Images were linearly adjusted for brightness and contrast using Adobe-Photoshop CS4 v.11 software.

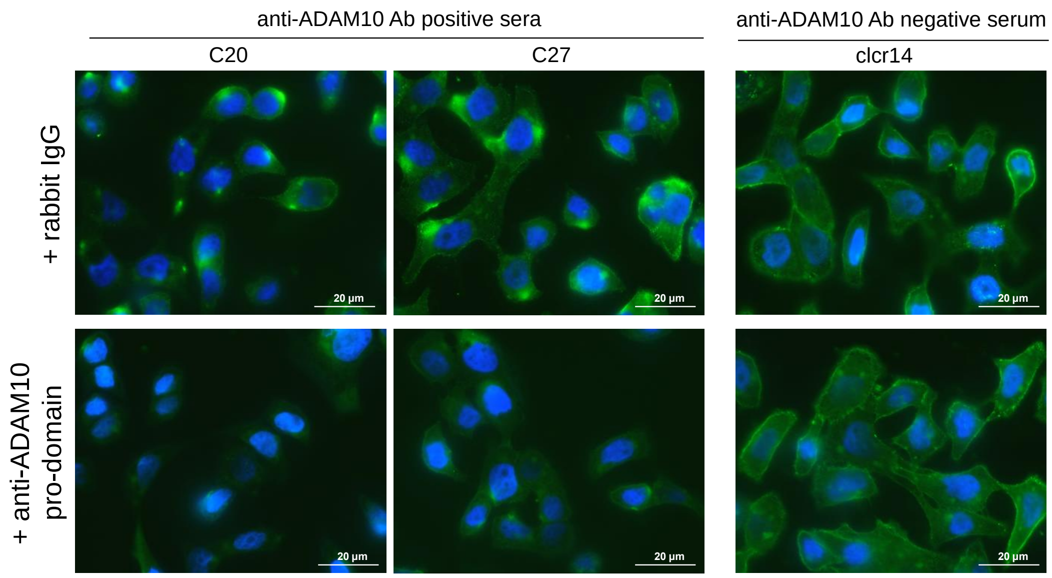

Supplemental Materials SM-Figure 5. *Immunofluorescence reactivity after competition with anti ADAM10 pro-domain Ab.* Anti-ADAM10 Ab positive sera: reactivity of the IgG fraction purified from representative sera of Crc patients (C20 and C27) considered positive for the presence of anti ADAM10 auto-Abs after competition either with control rabbit IgGs or the anti-ADAM10 pro-domain Ab. Anti-ADAM10 Ab negative serum: reactivity of the IgG fraction purified from the serum of the Crc patient (clcr14), considered negative for the presence of auto-Ab anti-ADAM10 but that showed a weak diffuse reactivity. Immunoreactivity was assessed after competition either with control rabbit IgGs or anti-ADAM10 pro-domain Ab. Cell nuclei were stained with Hoechst-33342; secondary Abs were goat anti-human IgG FITC. Images were acquired by immunofluorescence microscopy (Zeiss Upright Axo Imager 2 equipped with AxoVision Rel.4.8.2 software); magnification 63X. Images were linearly adjusted for brightness and contrast using Adobe-Photoshop CS4 v.11 software.

Supplemental Materials Table 1

Demographic and clinical features of the colorectal cancer patients testing cohort Crc1

Ptz #	Sex	Age	Stage (category)[a]	G	Tumor site	Neoad. therapy	Follow-up	Imm. hist.	Tumor lysate	anti-ADAM 10 OD	anti-ADAM 10 pos/neg
clcr65	m	53	Stg I (T1N0M0)	2	colon	-	A 24			228.52	-
clcr67	f	57	Stg I (T1N0M0)	1	colon	-	A 52			1157.94	+
clcr41	m	57	Stg I (T2N0M0)	1	colon	-	A 60			159.60	-
clcr32	m	83	Stg I (T2N0M0)	1	colon	-	A 33	y		242.94	+
clcr35	m	73	Stg I (T2N0M0)	2	colon	-	A 34			84.55	-
clcr66	f	61	Stg I (T2N0M0)	2	colon	-	A 53			46.34	-
clcr50	m	71	Stg I (T2N0M0)	2	colon	-	A 29			122.09	-
clcr71	m	66	Stg I (T2N0M0)	-	rectum	CT	-			390.35	+
clcr57	m	59	Stg II (T4N0M0)	2	colon	CT	A 59			691.37	+
clcr25	f	73	Stg II (T3N0M0)	2	colon	CT	A 34			364.93	+
clcr18	m	73	Stg II (T3N0M0)	2	colon	CT	M 6, †32	y	y	785.73	+
clcr26	f	60	Stg II (T3N0M0)	2	colon	CT	A 63	y		117.89	-
clcr4	f	53	Stg II (T3N0M0)	2	colon	CT	A 64			71.82	-
clcr42	m	84	Stg II (T3N0M0)	2	colon	CT	A 60			14.83	-
clcr45	m	80	Stg II (T3N0M0)	2	colon	CT	A 59	y	y	182.24	-
clcr56	f	57	Stg II (T3N0M0)	2	colon	CT	A 59			372.12	+
clcr38	m	81	Stg II (T3N0M0)	2	colon	CT	A 58			529.93	+
clcr27	m	61	Stg II (T3N0M0)	2	colon	CT	A 61			565.57	+
clcr1	f	51	Stg II (T3N0M0)	2	colon	CT	A 73			455.41	+
clcr6	f	63	Stg II (T3N0M0)	3	colon	CT	A 60			569.43	+
clcr46	f	62	Stg II (T3N0M0)	2	colon	CT	A 59			596.50	+
clcr24	f	75	Stg II (T3N0M0)	3	colon	CT	A 64			707.75	+
clcr14	f	70	Stg II (T4N0M0)	2	colon	CT	M 10, †28	y	y	25.42	-
clcr21	m	82	Stg II (T4N0M0)	3	colon	CT	A 64			1169.88	+
clcr7	m	74	Stg II (T3N0M0)	2	colon	CT	A 38			2098.17	+
clcr63	m	68	Stg III (T2N1M0)	-	colon	CT	-			301.18	+
clcr33	f	55	Stg III (T3N1M0)	2	colon	CT	†23		y	199.54	-
clcr2	f	61	Stg III (T3N1M0)	2	colon	CT	R 28, M 28, A 38			178.14	-
clcr15	f	68	Stg III (T3N1M0)	2	colon	CT	A 63	y	y	518.75	+
clcr40	f	69	Stg III (T3N1M0)	2	colon	CT	M 17			548.63	+
clcr23	m	79	Stg III (T3N1M0)	2	colon	CT	M 34			674.81	+
clcr29	m	80	Stg III (T3N1M0)	2	colon	CT	R 11, M 35	y	y	998.23	+
clcr43	m	73	Stg III (T3N1M0)	2	colon	CT	M 55, A 55			702.51	+
clcr3	m	67	Stg III (T3N1M0)	2	colon	CT	M 3, †21			843.53	+
clcr39	m	66	Stg III (T3N1M0)	2	colon	CT	A 33			1237.61	+
clcr22	f	74	Stg III (T3N1M0)	2	colon	CT	A 62	y		949.55	+
clcr16	m	90	Stg III (T3N1M0)	2	colon	CT	M 10, †30	y		1470.67	+
clcr36	m	81	Stg III (T3N2M0)	2	colon	CT	†14	y	y	59.94	-
clcr37	f	83	Stg III (T2N2M0)	2	colon	CT	M 26, †30		y	54.65	-
clcr69	m	58	Stg III (T2N2M0)	-	rectum	CT	-			766.55	+
clcr70	m	44	Stg III (T3N2M0)	-	colon	CT	-			510.18	+
clcr54	f	73	Stg III (T3N2M0)	2	colon	CT	M 2, †12	y	y	644.22	+
clcr11	m	64	Stg III (T3N2M0)	2	colon	CT	A 37			334.40	+
clcr68	m	69	Stg III (T3N2M0)	2	colon	CT	A 53			261.01	+
clcr28	f	44	Stg III (T3N2M0)	2	colon	CT	M 3, A 35	y		587.98	+
clcr51	m	66	Stg III (T3N2M0)	2	colon	CT	M 3, †27			834.34	+
clcr44	f	48	Stg III (T4N1M0)	3	colon	CT	M 23, A 31		y	3.91	-
clcr13	f	52	Stg III (T4N1M0)	3	colon	CT	-	y		369.47	+
clcr52	m	76	Stg III (T4N2M0)	2	colon	CT	M 6, †10			351.84	+
clcr20	m	45	Stg III (T4N2M0)	2	colon	CT	†28	y		757.57	+
clcr49	f	77	Stg III (T4N2M0)	2	colon	CT	A 29			511.99	+
clcr12	f	60	Stg IV (T4N1M1)	3	colon	CT	R 14, M 16, A 24			319.54	+
clcr17	f	65	Stg IV (T4N2M1)	3	colon	CT	†15	y		303.44	+
clcr19	m	74	Stg IV (T4N2M1)	3	colon	CT	†8	y	y	941.90	+
clcr8	f	66	Stg IV (metas [b])	-	colon	CT	†6			962.58	+
clcr48	m	66	Stg IV (metas)	-	colon	CT	R 10, †17			109.39	-
clcr55	m	57	Stg IV (metas)	-	colon	CT	†8			479.18	+

a = staging and category of colon carcinoma according to TNM classifications. UICC, tumor, nodes, metastases. T1-T4, invasive tumors (T1, submucosa; T2, tunica muscularis; T3, subserosa; T4, peritoneum or other organs). N0, no malignant regional lymphnodes; N1, 1-3 regional lymphnodes metastases; N2, >4 regional lymphnode metastases. M0, no distant metastases; M1, distant metastasis. Stage I (T1-2, N0, M0), Stage II (T3-4, N0, M0), Stage III (T1-4, N1-2, M0), Stage IV (any T, any N, M1).

b = liver metastasis of colon carcinoma; m= male; f= female. OD= normalized optical density (arbitrary units); G= grading; Imm. hist.= immuno histochemistry; pos/neg = positive and negative sera reactivity according to the 237 OD cut-off defined by ROC analysis. Neoad.= neoadjuvant; CT= chemotherapy; RT= radiotherapy; A = alive; M= metastasis; R= relapse; †= dead.

Supplemental Materials Table 2

Demographic and clinical features of the colorectal cancer patients validation cohort Crc2

Ptz #	Sex	Age	Stage (category)[a]	G	Tumor site	Neoad. therapy	Follow-up	Imm. hist.	Tumor lysate	anti-ADAM 10	
										OD	pos/neg
C7	f	72	Stg I (T1N0M0)	1	colon	-	A 47			819.88	+
C12	m	41	Stg I (T1N0M0)	3	rectum	CT	A 46		y	85.37	-
C15	m	71	Stg I (T1N0M0)	2	rectum	CT	A 6			399.89	+
C42	f	60	Stg I (T1N0M0)	1	rectum	CT/RT	A 43			1493.09	+
C48	m	65	Stg I (T1N0M0)	1	colon	-	A 41			625.58	+
C1	m	78	Stg I (T2N0M0)	2	colon	-	A 47			152.42	-
C6	f	54	Stg I (T2N0M0)	2	colon	-	-			1044.75	+
C13	m	77	Stg I (T2N0M0)	1	colon	-	A 26			1.05	-
C14	f	85	Stg I (T2N0M0)	2	colon	-	-			1211.66	+
C24	f	73	Stg I (T2N0M0)	2	colon	-	A 45			408.69	+
C29	f	70	Stg I (T2N0M0)	2	colon	-	A 44			137.49	-
C38	m	85	Stg I (T2N0M0)	3	colon	-	A 37	y	y	284.23	+
C43	m	50	Stg I (T2N0M0)	2	rectum	CT/RT	A 42		y	243.32	+
C3	m	59	Stg II (T3N0M0)	2	colon	CT	-		y	127.31	-
C9	f	77	Stg II (T3N0M0)	2	colon	CT	A 47	y	y	675.95	+
C11	f	72	Stg II (T3N0M0)	2	colon	CT	-		y	70.68	-
C18	m	84	Stg II (T3N0M0)	2	colon	CT	A 45			478.16	+
C19	m	64	Stg II (T3N0M0)	2	colon	CT	A 25	y		1068.60	+
C21	f	79	Stg II (T3N0M0)	2	colon	CT	A 45		y	458.16	+
C23	f	74	Stg II (T3N0M0)	2	colon	CT	A 45			398.35	+
C27	m	80	Stg II (T3N0M0)	3	colon	CT	A 44		y	1047.59	+
C30	m	78	Stg II (T3N0M0)	2	colon	CT	A 44		y	551.13	+
C37	f	78	Stg II (T3N0M0)	3	colon	CT	A 43	y		3003.21	+
C39	f	96	Stg II (T3N0M0)	3	colon	CT	A 43	y		773.40	+
C44	f	52	Stg II (T3N0M0)	2	colon	CT	A 43	y		279.52	+
C49	m	72	Stg II (T3N0M0)	2	colon	CT	A 41			267.58	+
C36	f	62	Stg III (T2N1M0)	3	rectum	CT	A 43		y	1293.03	+
C5	m	47	Stg III (T3N1M0)	2	rectum	CT	R 43, A 47			648.04	+
C20	m	70	Stg III (T3N1M0)	3	colon	CT	A 45		y	1818.22	+
C22	m	49	Stg III (T3N1M0)	2	colon	CT	A 45			695.34	+
C26	m	70	Stg III (T3N1M0)	2	rectum	CT	R 16, A 45	y	y	199.53	-
C31	m	73	Stg III (T3N1M0)	2	colon	CT	A 44			352.80	+
C40	f	80	Stg III (T3N1M0)	2	colon	CT	†23			767.00	+
C46	f	65	Stg III (T3N1M0)	3	colon	CT	A 41			308.26	+
C4	f	69	Stg III (T4N1M0)	3	colon	CT	A 47	y	y	595.12	+
C16	f	77	Stg III (T4N1M0)	2	colon	CT	A 46			3213.93	+
C41	f	70	Stg III (T3N2M0)	2	rectum	CT	R 14, A 43		y	875.97	+
C47	f	67	Stg III (T3N2M0)	1	rectum	CT	A 41			350.17	+
C51	m	56	Stg III (T3N2M0)	3	colon	CT	A 40			240.41	+
C10	m	64	Stg IV (T3N1M1)	-	colon	CT	R 29, †29			637.29	+
C8	m	45	Stg IV (T3N0M1)	2	rectum	CT	A 47			970.28	+
C28	m	37	Stg IV (T3N0M1)	2	rectum	CT	M 10, †27			256.70	+
C25	m	55	Stg IV (T1N1M1)	3	colon	CT	A 25	y		99.39	-
C17	f	61	Stg IV (T3N1M1)	2	colon	CT	R 15	y		695.89	+
C35	f	72	Stg IV (T3N1M1)	2	colon	CT	R 7, A 23		y	192.70	-
C45	m	68	Stg IV (T3N1M1)	2	colon	CT	R 29, A 43			94.87	-
C2	m	67	Stg IV (T4N1M1)	2	colon	CT	R 26, A 43			2985.07	+
C32	f	72	Stg IV (T4N1M1)	2	colon	CT	†28			94.93	-
C52	m	75	Stg IV (T3N2M1)	2	colon	CT	R 6, †26		y	125.84	-

a = staging and category of colon carcinoma according to TNM classifications. UICC, tumor, nodes, metastases. T1-T4, invasive tumors (T1, submucosa; T2, tunica muscularis; T3, subserosa; T4, peritoneum or other organs). N0, no malignant regional lymphnodes; N1, 1-3 regional lymphnodes metastases; N2, >4 regional lymphnode metastases. M0, no distant metastases; M1, distant metastasis. Stage I (T1-2, N0, M0), Stage II (T3-4, N0, M0), Stage III (T1-4, N1-2, M0), Stage IV (any T, any N, M1).

b = liver metastasis of colon carcinoma; m= male; f= female. OD= normalized optical density (arbitrary units); G= grading; Imm. hist.= immuno histochemistry; pos/neg = positive and negative sera reactivity according to the 237 OD cut-off defined by ROC analysis. Neoad.= neoadjuvant; CT= chemotherapy; RT= radiotherapy; A = alive; M= metastasis; R= relapse; †= dead.

Supplemental Materials Table 3
Demographic and clinical features of the control subjects (Cn1) included in the testing cohort

				anti-ADAM 10 reactivity	
**Subject #**	**Gender**	**Age**	**Clinical status**	OD	pos/neg
1	f	79	inguinal hernia	2.48	-
2	f	59	endometrial polyp	3.13	-
3	f	68	hepatic cyst	365.42	+
4	m	44	healthy	39.74	-
5	m	45	healthy	39.74	-
6	f	61	diverticulosis	261.29	+
7	f	73	cataract	80.05	-
8	m	71	cataract	25.93	-
9	f	76	rectocele	78.27	-
10	f	58	hyperparathyroidism	110.31	-
11	m	69	cirrhosisr	103.69	-
12	m	86	diabetes	420.81	+
13	m	69	rectal prolapse	199.16	-
14	m	73	inguinal hernia	323.74	+
15	m	81	pulmonary embolism	125.02	-
16	f	43	healthy	177.42	-
17	f	76	diabetes	130.37	-
18	f	27	healthy	176.83	-
19	f	58	healthy	125.49	-
20	m	69	healthy	176.11	-
21	m	73	healthy	228.38	-
22	f	50	cervical ectropion	351.28	+
23	f	58	healthy	306.22	+
24	f	57	thyroid goiter	233.93	-
25	f	70	diverticulosis	233.29	-
26	m	79	healthy	407.87	+
27	m	81	healthy	473.58	+
28	f	73	healthy	312.29	+
29	f	64	bronchial asthma	1.60	-
30	f	67	healthy	442.75	+
31	m	77	inguinal hernia	186.24	-
32	f	26	healthy	477.16	+
33	m	91	hemorroiditis	27.47	-
34	f	62	healthy	558.21	+
35	f	73	diabetes	515.14	+
36	f	30	healthy	126.18	-
37	f	67	healthy	734.00	+
38	m	60	healthy	127.21	-
39	f	61	cholecystitis	553.93	+

m: male; f: female. OD= optical density (arbitrary units);
pos/neg = positive and negative sera reactivity according to the 237 OD cut-off defined by ROC analysis.

Supplemental Materials Table 4

Demographic and clinical features of the control subjects (Cn2) included in the validation cohort

				anti-ADAM 10 reactivity	
**Subject #**	**Sex**	**Age**	**Clinical status**	OD	pos/neg
40	m	74	healthy	202.49	-
41	m	57	healthy	888.54	+
42	m	76	healthy	262.00	+
43	f	65	healthy	58.78	-
44	m	82	healthy	91.88	-
45	m	53	healthy	618.80	+
46	m	76	healthy	353.64	+
47	m	66	healthy	183.68	-
48	f	77	healthy	220.25	-
49	m	76	healthy	85.67	-
50	m	67	healthy	86.21	-
51	m	52	healthy	528.48	+
52	m	69	healthy	827.93	+
53	m	72	healthy	968.50	+
54	f	88	healthy	226.19	-
55	f	70	healthy	169.61	-
56	m	67	healthy	60.88	-
57	f	67	healthy	303.60	+
58	m	72	healthy	228.13	-
59	m	61	healthy	662.79	+
60	f	51	healthy	3.43	-
61	f	59	healthy	202.44	-
62	f	53	healthy	39.32	-
63	f	60	healthy	144.45	-
64	f	75	healthy	160.31	-
65	f	57	healthy	195.97	-
66	f	76	healthy	173.15	-
67	f	91	healthy	204.18	-
68	f	57	healthy	446.88	+
69	f	52	healthy	566.61	+
70	f	63	healthy	3.75	-
71	f	92	healthy	221.09	-
72	f	55	healthy	42.94	-
73	f	68	healthy	157.77	-
74	f	89	healthy	175.08	-
75	f	78	healthy	214.03	-
76	m	45	healthy	431.76	+
77	m	32	healthy	102.86	-
78	f	68	healthy	533.68	+
79	f	51	healthy	150.10	-
80	f	55	healthy	552.64	+
81	m	45	healthy	108.71	-
82	f	48	healthy	370.80	+
83	f	44	healthy	489.91	+
84	f	69	healthy	112.27	-
85	m	45	healthy	84.39	-
86	m	54	healthy	615.50	+
87	f	70	healthy	332.42	+
88	f	34	healthy	290.05	+
89	m	49	healthy	137.49	-
90	f	59	healthy	104.45	-
91	m	71	healthy	159.56	-

m: male; f: female. OD= optical density (arbitrary units);
pos/neg = positive and negative sera reactivity according to the 237 OD cut-off defined by ROC analysis.

Supplemental Materials Table 5

Summary of the demographic features and the anti-ADAM 10 serological reactivity of the tumors of epithelial and non-epithelial origin

			anti-ADAM 10 reactivity						
	N (m/f)	Age	normalized OD	95% CI	OD *vs.* Cn (M-W)	237 OD cut-off	Fischer's test	sens.	spec.
Cn	91 (37/54)	63.88 sd 14.24	261.7 se 22.2	217.5-306	-	34+ 57-	-	-	0.63
Pc	43 (23/20)	66.77 sd 9.75	727.5 se 127.5	470.2-984.8	p=0.0006	31+ 12-	p=0.0002	0.72	-
Brc	43 (0/43)	53.40 sd 11.02	436.1 se 45.58	344.1-528	p=0.0003	30+ 13-	p=0.0008	0.70	-
B-CLL	53 (34/19)	65.43 sd 10.76	318.7 se 35.11	248.3-389.2	ns	28+ 25-	ns	0.53	-
MM	46 (21/25)	66.33 sd 10.48	266.5 se 36.8	192.4-340.6	ns	21+ 25-	ns	0.46	-

Cn= control subjects cohort 1+2; Pc= pancreatic carcinoma; Brc= breast carcinoma; B-CLL= B-cell chronic lymphocytic leukemia; MM=multiple myeloma; m= male; f= female; sd= standard deviation; se= standard error; OD= optical density; 95% CI= 95% confidence interval; M-W= Mann-Whitney test; 237 OD cut-off= sera considered positive (+) or negative (-) according to the 237 OD value defined for colorectal cancer; sens.= sesitivity; spec.= specificity.

Supplemental Materials Table 6

ADAM10 expression and inflammatory lymphoid cells infiltrate evaluation by immunohistochemistry in the Crc patients' tumor

Patient #	ADAM10 /score	Tumoral stage (TNM)	Inflammatory infiltrate	anti-ADAM 10	
				normalized OD	reactivity
clcr32	±/1	I (T2N0M0)	2	242.9	+
C38	+/2	I (T2N0M0)	3	284.23	+
clcr18	±/1	II (T3N0M0)	1	785.73	+
clcr26	±/1	II (T3N0M0)	2	117.89	-
clcr45	±/1	II (T3N0M0)	2/3	182.24	-
clcr14	±/1	II (T4N0M0)	1	25.42	-
C9	+/3	II (T3N0M0)	2	675.95	+
C19	+/3	II (T3N0M0)	1	1068.60	+
C37	+/3	II (T3N0M0)	2	3003.21	+
C39	+/2	II (T3N0M0)	2	773.40	+
C44	+/3	II (T3N0M0)	2	279.52	+
clcr15	±/1	III (T3N1M0)	2/3	518.75	+
clcr29	±/1	III (T3N1M0)	1	998.23	+
clcr22	+/1	III(T3N1M0)	2	949.55	+
clcr16	+/1	III (T3N1M0)	1/2	1470.67	+
C26	+/1	III (T3N1M0)	1	199.53	-
clcr36	+/3	III (T3N2M0)	1	59.94	-
clcr54	+/3	III (T3N2M0)	1	644.22	+
clcr28	±/1	III (T3N2M0)	3	587.98	+
clcr13	±/1	III (T4N1M0)	1	369.47	+
clcr20	±/1	III (T4N2M0)	1	757.57	+
C4	+/3	III (T4N1M0)	2	595.12	+
clcr17	±/1	IV (T4N2M1)	1	303.44	+
clcr19	+/2	IV (T4N2M1)	1	941.90	+
C25	+/1	IV (T1N1M1)	2	99.39	-
C17	+/3	IV (T3N1M1)	3	695.89	+

score= number of positive cells or clusters (1=low; 2=medium; 3=high); Inflammatory infiltrate: 1=scarce; 2=medium; 3=high; OD= optical density (arbitrary units).
